# Molecular Mechanisms Underlying the Effects of Bimin Kang Mixture on Allergic Rhinitis: Network Pharmacology and RNA Sequencing Analysis

**DOI:** 10.1155/2022/7034078

**Published:** 2022-10-28

**Authors:** Li-Jie Qi, Ren-Zhong Wang, Shang Gao, Xiang-Jing Chen, Xin Zhang, Yi-Peng Zhang

**Affiliations:** ^1^The First Clinical Medical College, Shandong University of Traditional Chinese Medicine, Jinan, Shandong 250355, China; ^2^Department of Otorhinolaryngology, Affiliated Hospital of Shandong University of Traditional Chinese Medicine, Jinan, Shandong 250014, China

## Abstract

**Background:**

Allergic rhinitis (AR) is a highly prevalent chronic inflammatory disease of the respiratory tract. Previous studies have demonstrated that Bimin Kang Mixture (BMK) is effective in alleviating AR symptoms and reducing the secretion of inflammatory factors and mucin; however, the precise mechanisms underlying these effects remain unclear.

**Methods:**

We built target networks for each medication component using a network pharmacology technique and used RNA-seq transcriptome analysis to screen differentially expressed genes (DEGs) for AR patients and control groups. The overlapping targets in the two groups were assessed using PPI networks, GO, and KEGG enrichment analyses. The binding ability of essential components to dock with hub target genes was investigated using molecular docking. Finally, we demonstrate how BMK can treat AR by regulating the NF-*κ*B signaling pathway through animal experiments.

**Results:**

Effective targets from network pharmacology were combined with DEGs from RNA-seq, with 20 intersections as key target genes. The construction of the PPI network finally identified 5 hub target genes, and all hub target genes were in the NF-*κ*B signaling pathway. Molecular docking suggests that citric acid, deoxyandrographolide, quercetin, luteolin, and kaempferol are structurally stable and can spontaneously attach to IL-1*β*, CXCL2, CXCL8, CCL20, and PTGS2 receptors. Animal experiments have shown that BMK inhibits NF-*κ*B transcription factor activation, reduces the expression of proinflammatory cytokines and chemokines IL-1*β*, CXCL2, IL-8, and COX-2, and exerts anti-inflammatory and anti-allergic effects.

**Conclusion:**

BMK by regulating the NF-*κ*B signaling pathway improves inflammatory cell infiltration, regulates mucosal immune balance, and reduces airway hypersensitivity. These findings provide theoretical support for the clinical efficacy of BMK for AR treatment.

## 1. Introduction

Allergic rhinitis (AR) is a noninfectious inflammatory disease of the nasal mucosa mediated by immunoglobulin E (IgE), developing after an atopic individual is exposed to an allergen. The typical clinical manifestations of AR include nasal congestion, nasal itching, mucosal drainage, and sneezing [1]. Epidemiological surveys have demonstrated that the global prevalence of AR ranges from approximately 10 to 40%, and these rates continue to increase each year [2]. AR is often accompanied by other diseases such as conjunctivitis, rhinosinusitis, and otitis media and substantially increases the risk of developing asthma [3]. Indeed, at least 60% of patients with asthma exhibit AR, while 20–30% of patients with AR have been diagnosed with asthma [4]. Thus, in addition to potentially severe effects on health and quality of life, AR is associated with a significant economic burden worldwide [5].

Currently, common treatments for AR include reducing allergen exposure, drug therapy, allergen-specific immunotherapy (AIT), and biologics [6]. Drug therapy includes oral or topical H1-antihistamines, leukotriene receptor antagonists, anticholinergics, decongestants, and intranasal corticosteroids. However, multidrug combinations and treatment regimens that target symptoms do not address the underlying causes of AR. Although AITs including subcutaneous immunotherapy (SCIT) and sublingual immunotherapy (SLIT) can achieve long-term efficacy via modulation of the immune system [7–9], this option is less than ideal given the long treatment cycles required and poor adherence among patients. Simpler, more effective regimens that involve fewer drugs and lead to fewer side effects are therefore desirable.

Chinese herbal medicine has been widely used in the clinical treatment of AR and plays an important role in alleviating symptoms and controlling inflammation. Bimin Kang Mixture (BMK) contains 18 herbal medicines, which is a formula based on two classic prescriptions, Yupingfeng San (YPFS) and Xiaoqinglong Decoction (XQLD), to which other Chinese herbs are added or subtracted (Table 1).

When the body is exposed to an allergen, immunoglobulin E binds to high-affinity receptors (Fc*ε*RI) on the surface of mast cells, inducing a sensitized state. When exposed to the allergen again, mast cells release chemical mediators such as histamine, triggering a series of inflammatory responses [10]. Previous studies have demonstrated that YPFS can control inflammation by inhibiting the activation of mast cells, thereby exerting anti-inflammatory and immunomodulatory effects [11–13]. In addition, XQLD has been shown to exhibit antihistaminergic and anti-inflammatory effects, helping to alleviate symptoms and safely improve quality of life in patients with AR. Animal studies have also shown that XQLD inhibits aryl hydrocarbon receptor (AHR) and reduces airway inflammation and remodeling [14]. Another study confirmed that XQLD reduced levels of inflammatory factors such as IgE, IL-4, and IL-13 in the nasal mucosa while restoring the balance of Th1/Th2 cells and the integrity of the mucosal structure [15].

BMK combines YPFS, XQLD, and several other Chinese herbs into one prescription, doubling treatment efficacy and addressing the fundamental cause of AR. Our previous studies have verified the clinical efficacy of BMK, highlighting its potential to significantly attenuate symptoms and improve quality of life in patients with AR [16, 17]. Animal experiments have also demonstrated that BMK can effectively reduce the infiltration of eosinophilic inflammatory cells into the nasal mucosa and reduce the secretion of mucin 5 subtype AC (MUC5AC) and mucin 5 subtype B (MUC5B) into the serum, which helps to improve AR symptoms such as runny-nose [18]. In addition, BMK can reduce the secretion of IL-6, IL-8, and tumor necrosis factor alpha (TNF-*α*), thereby mitigating the inflammatory response. Research has also indicated that BMK induces relative increases in the CD3+ and CD4+ expression and a decrease in the CD8 + expression, further demonstrating its immunomodulatory function [19].

The Chinese herbal formulae are characterized by diverse components and complex biological relationships, and similarly, allergic rhinitis involves the interaction of multiple target genes and functional proteins. Network pharmacology is a research tool that can construct complex synergistic relationships between drug components, disease-related targets, and molecular pathways to comprehensively assess the potential therapeutic effects of herbal formulations on the disease through multichannel modulation of signaling pathways [20, 21]. In addition, high-throughput RNA-seq, the method of choice for studying differential gene expression, has a high resolution for differential gene expression levels [22].

**Figure 1:**
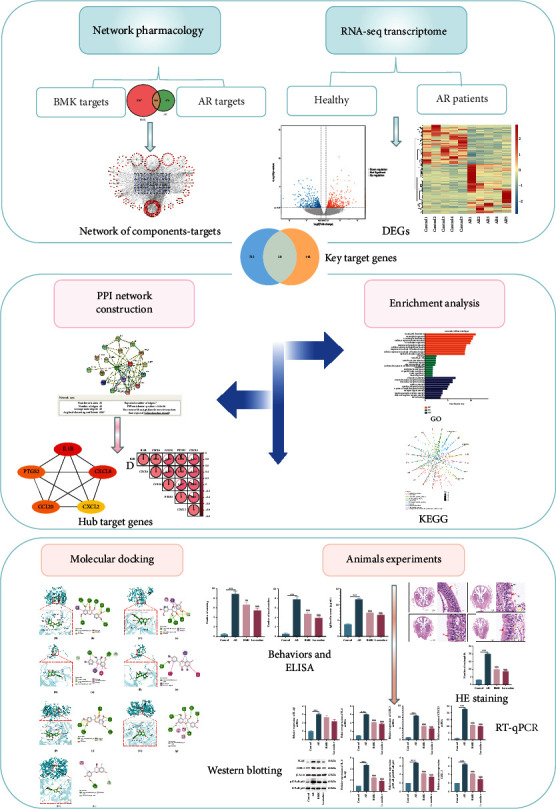
Based on network pharmacology and RNA-SEQ to explore the mechanism of BMK in the treatment of AR.

BMK contains numerous herbal components, and the specific anti-inflammatory and immunomodulatory mechanisms associated with its efficacy in the context of AR remain unclear. Therefore, we utilized network pharmacology and RNA-seq to provide evidence supporting the clinical application of BMK by performing a comprehensive analysis of its active components, potential targets, and relevant mechanisms of action in the context of AR (Figure 1).

## 2. Materials and Methods

### 2.1. Identification of Effective BMK Components

Most of the chemical components of BMK were obtained from the Traditional Chinese Medicine Systems Pharmacology (TCMSP) platform (https://old.tcmsp-e.com/tcmsp.php) [23]. Effective components of each drug in BMK were screened using oral bioavailability (OB) ≥ 30% and drug − likeness (DL) ≥ 0.18 as the criteria. The gene names of the relevant target proteins obtained from the TCMSP database were normalized using UniProt (https://www.uniprot.org/) [24]. Since the effective components of *Pheretima* and *Cicadae Periostracum* could not be retrieved from the TCMSP database, they were identified using SymMap (version 2.0) [25].

### 2.2. Identification of AR-Related Target Genes

Target genes related to AR were identified by searching the following databases for the key term “allergic rhinitis”: Therapeutic Target Database (TTD) (http://db.idrblab.net/ttd/), DrugBank (https://go.drugbank.com/), GeneCards (https://www.genecards.org/), and Online Mendelian Inheritance in Man (OMIM) (https://www.omim.org/) [26–29]. Statistical results are presented in a Venn diagram [30].

### 2.3. Construction of the Effective Component–Target Gene Network

The two target genes identified in the previous step were linked, and the duplicated region was considered an effective target for BMK in the treatment of AR. The overlap is represented as a Venny (version 2.1.0) diagram. Cytoscape software (version 3.7.2) [31] was used to construct a network of therapeutic target genes corresponding to the active components of the Chinese herbal medicines.

### 2.4. RNA Sequencing Analysis

Patients who satisfied the diagnostic criteria for AR were included in this study, according to the guidelines for the diagnosis and treatment of AR [1, 32]. Specific diagnostic criteria were as follows. symptoms: nasal congestion, nasal itching, sneezing, and runny nose appear ≥2 times, and the symptoms last for ≥1 hour every day or may be accompanied by itchy eyes, tears, or red eyes; signs: pale and edema of the nasal mucosa and watery secretion in the nasal cavity; and allergen test: at least one skin allergy origin prick test is positive. The current study included healthy controls (*n* = 5) and patients with AR (*n* = 5). The research plan was approved by the Ethics Committee of the Affiliated Hospital of Shandong University of Traditional Chinese Medicine (approval no. [2020] No. [044] to KY), and all participants provided written informed consent. Nasal epithelial cells were collected from each participant for RNA-seq using nasal swabs. First, total RNA was extracted from the collected samples using an RNAsimple Total RNA Kit 50 (Tiangen, China, DP419). An Agilent Bioanalyzer 2100 (Agilent Technologies, Santa Clara, CA, US) was used to verify RNA integrity. The purity and concentration of the total RNA were measured using a Qubit ® 3.0 Fluorometer (Life Technologies, CA, USA) and Nanodrop One spectrometer (Thermo Fisher Scientific Inc., USA). A library was constructed for the samples that met the detection criteria, following which mRNA was detected using an Illumina NovaSeq 6000 sequencer. (The testing process was carried out by Shanghai Jingzhou Gene Technology Co., Ltd.)

### 2.5. Identification of Differentially Expressed Genes (DEGs)

The Edge*R* software package was used to analyze DEGs among the two groups (control vs. AR). Simultaneously, the differential expression multiple (fold change, FC) was calculated according to the fragments per kilobase of exon per million reads mapped (FPKM) value, which is often represented as log_2_|FC|. Finally, due to the small number of DEGs obtained via screening, the FC threshold standard was appropriately adjusted to up 1.5 times or down 0.67 times, *P* < 0.05. DEGs were visualized using volcano plots and heat maps.

### 2.6. Functional Enrichment and Pathway Analysis

The Gene Ontology (GO) [33] and Kyoto Encyclopedia of Genes and Genomes (KEGG) [34, 35] databases are often used to clarify the biological functions and signaling pathways associated with specific genes *in vivo* or *in vitro*. The GO database defines genes and proteins based on cellular components (CC), biological processes (BP), and molecular functions (MF). GO enrichment analysis was performed using a *P* value <0.05 as the threshold. The KEGG database contains information related to genomic, chemical, and system functions and graphically illustrates the signaling pathways involved in the target genes. In this study, the Database for Annotation, Visualization, and Integrated Discovery (DAVID) (version 6.8) (https://david.ncifcrf.gov/) [36] was used to functionally annotate the therapeutic targets of BMK in the context of AR and to perform enrichment analysis of key target genes.

### 2.7. Construction of the PPI Network

PPI play a key role in disease, and PPI network analysis can aid in identifying the molecular mechanisms underlying disease process as well as new molecular targets [37].The STRING database (https://cn.string-db.org/) [38] was used to construct the PPI network, which was set using a minimum required interaction score of 0.400 and hidden disconnected nodes as parameters. Subsequently, Cytoscape (version 3.7.2) was used for visual analysis. Topology analysis was evaluated based on calculated network parameter results, including closeness centrality (CC), betweenness centrality (BC), topological coefficient (TC), and degree centrality (DC). Sorting was performed according to the highest degree value, and the top five genes were selected as hub target genes.

### 2.8. Molecular Docking

According to the degree value of the network construction, the five active ingredients with the highest scores were selected for molecular docking with the hub target genes. First, we obtained receptor and ligand structures from PubChem (https://pubchem.ncbi.nlm.nih.gov/) and RCSB PDB (https://www.rcsb.org/) databases. Then, the ligand energy was minimized using Chem 3D (version 20.0), and the receptor was treated with dehydration and residue removal by PyMol software (version 1.4.1). After that, AutoDock Tools (version 1.5.7) was used to convert the files into (.pdbqt) format for molecular docking, the structure was evaluated by AutoDock Vina software (version 1.2.0), and the lower the affinity energies, the more stable the structure. Finally, visualization was performed using PyMol and Discovery Studio 2021 software.

### 2.9. Animal Experiments

Female specific pathogen free (SPF) BALB/C mice (*n* = 36, age: 6-8 weeks, weight: 18-20 g) were purchased from Jinan Pengyue Experimental Animal Breeding Co., Ltd. (Shandong, China; animal license number: SCXK (Lu) 20190003). The dose utilized in the present study was based on the results of our previous experiments, which verified the optimal efficacy of the medium dose [18]. The mice were randomly divided into four groups (*n* = 6 in each): control, AR, BMK, and loratadine. The animals were raised at the Animal Experimental Center of the Affiliated Hospital of Shandong University of Traditional Chinese Medicine (temperature: 23 ± 2°C, relative humidity: 55 ± 10%, 12 h light/dark cycle). Drinking water and food were provided *ad libitum*. The mice were modeled after 1 week of quarantine and adaptive feeding. All animal studies were approved by the Institutional Animal Care and Use Committee (IACUC) of Shandong University of Traditional Chinese Medicine (approval number: 2020-55).

**Figure 2:**
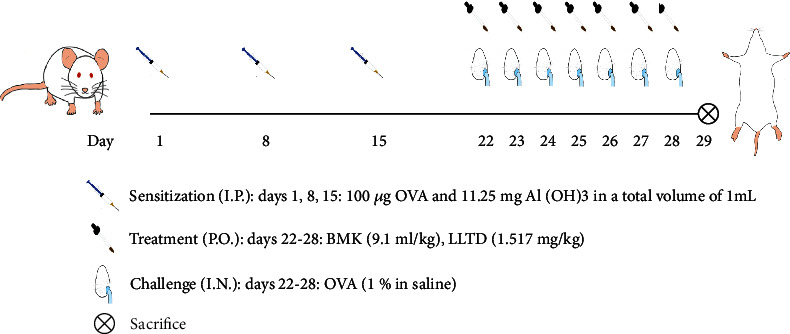
Schematic diagram of AR mice model construction and treatment process.

A mice model of AR was established using ovalbumin (OVA, Sigma Aldrich, USA, A5503) [39–43]. At the basic sensitization stage, the AR, BMK, and loratadine groups received intraperitoneal injections of 200 *μ*L of the sensitization drugs (100 *μ*g/ml OVA and 11.25 mg/ml aluminum hydroxide (Al(OH)3; Sigma-Aldrich, USA, 239186, mixed suspension) on days 1, 8, and 15, respectively. The control group was intraperitoneally injected with the same volume of phosphate-buffered saline (PBS). On days 22 to 28, the AR, BMK, and loratadine groups received 1% inhaled OVA (10 mg/ml) for 20 min daily for 1 week. Simultaneously, the drug was administered intragastrically 1 h before OVA stimulation. The diluted drug concentration was determined using the body surface area method, and the BMK and loratadine groups were treated with a 200 *μ*L BMK diluent (9.1 ml/kg) (Affiliated Hospital of Shandong University of Traditional Chinese Medicine, approval number: Lu Medicine manufacturing Z01080047) and 200 *μ*L loratadine tablets (Clarityne, Shanghai Schering-Plough Pharmaceutical Co., Ltd., National Drug approval H10970410) dissolved in liquid (1.517 mg/kg), respectively. The blank and AR groups were administered 200 *μ*L PBS via gavage (Figure 2).

Twenty-four hours after the last OVA challenge, the mice were anesthetized via an intraperitoneal injection of 0.3% pentobarbital sodium (0.1 ml/10 g), following which the eyeball was removed for blood collection. The collected blood was centrifuged to obtain serum for enzyme-linked immunosorbent assays (ELISA), which was stored at -80°C. The nasal bone and maxilla were completely removed for hematoxylin and eosin (HE) staining. In other mice, the nasal mucosa was stripped for real-time quantitative polymerase chain reaction (RT-qPCR) and western blotting.

### 2.10. Evaluation of Nasal Symptoms

Thirty minutes after the last OVA challenge on day 28, the number of sneezing episodes and nasal scratches over a 10 min period was calculated by three blinded observers to assess improvements in nasal symptoms in AR mice treated with BMK.

### 2.11. ELISA

Total IgE levels in serum were determined using an ELISA kit (Mmbio, China, MM-0056M1), in accordance with the manufacturer's instructions. Four samples were taken from each group, and each assay was repeated three times.

### 2.12. HE Staining

The complete nasal structure was first soaked in 4% paraformaldehyde (Servicebio, China, G1101) and fixed for 24 h, following which it was placed in ethylenediaminetetraacetic acid (EDTA) decalcification solution (Servicebio, China, G107) for decalcification, embedded in paraffin, and cut into 5 *μ*m sections. The sections were then stained with HE (Servicebio, China, G1003), dehydrated, and sealed for further analysis. The structure of the nasal mucosa was observed under a light microscope, and three fields of view were randomly selected to assess the infiltration of eosinophils in each group.

### 2.13. RT-qPCR Analysis

First, total RNA was extracted from mice nasal mucosa using a TaKaRa extraction kit (TaKaRa, China, 9767), and the quality (concentration and purity) of total RNA was determined using an Ultramicro spectrophotometer (NanoDrop, USA, ND-1000). Total RNA was then reverse-transcribed into cDNA using a TaKaRa reverse transcription kit (TaKaRa, China, RR036A). Reference to the NCBI database (https://www.ncbi.nlm.nih.gov/tools/primer-blast/) was used for primer design, which was performed by Qinke Biotechnology (Beijing) Co., Ltd. Finally, TaKaRa (TaKaRa, China, RR820A) was used for real-time PCR detection. Using *β*-actin as an internal reference, the relative levels of the mRNA expression for interleukin-1B (IL-1B/IL-1*β*), C-X-C motif chemokine ligand 8 (CXCL8/IL-8), C-X-C motif chemokine ligand 2 (CXCL2/MIP-2), and prostaglandin-endoperoxide synthase 2 (PTGS2/COX-2) genes were calculated using the 2^-∆∆CT^ method. Primer sequences are shown in (Table 2).

### 2.14. Western Blotting

Protein levels were determined by western blotting. To extract total protein from mice nasal mucosal tissues, we employed RIPA lysate (Beyotime, Shanghai, P0013B) and PMSF (Beyotime, Shanghai, ST506). Before denaturation, protein concentration was determined using a BCA protein concentration determination kit (Beyotime, Shanghai, P0010S). To prepare a 10% gel for electrophoresis, an SDS-PAGE gel kit (Beyotime, Shanghai, P0012AC) was utilized. After blocking in 5% nonfat milk, antibodies were incubated: Anti-IL-1*β* (Abcam, 1 : 1000, ab283818), Anti-COX-2 (Proteintech, 1 : 3000, 66351-1-Ig), Anti-p-NF-*κ*B p65 (Abcam, 1 : 1000, ab239882), Anti-NF-*κ*B p65 (Abcam, 1 : 1000, ab16502), and Anti-*β*-actin (Abcam, 1 : 1000, ab16502) (Proteintech, 1 : 5000, 66009-1-Ig). Finally, strip development is carried out. ImageJ was used to examine the band density.

### 2.15. Statistical Analyses

SPSS (version 26.0) software was used for statistical analysis. The degree of correlation between variables was expressed by Pearson correlation coefficient. The differences between groups conformed to a normal distribution and were analyzed using one-way analyses of variance (ANOVA). The least significant difference (LSD) *t*-test was used for pairwise comparisons between groups. The results are presented as the mean ± standard error (SEM), and *P* values <0.05 were considered statistically significant (*P* < 0.01, obvious difference; *P* < 0.001, very significant difference; ns: no statistical significance). All statistical graphs were generated using GraphPad Prism (8.0).

## 3. Results

### 3.1. Effective Components and Target Genes of BMK

Candidate targets of 16 Chinese herbal medicines were obtained from the TCMSP database, and 257 components were screened according to their conditions (OB ≥ 30% and DL ≥ 0.18). According to the SymMap database, the numbers of effective components in *Pheretima* and *Cicadae Periostracum* were 19 and 29, respectively. In the process of searching for targets corresponding to each component, we noticed that some components had no relevant targets. Thus, these invalid components are not shown in the final chart. According to our statistical analysis, 244 effective components and 1,435 target genes in BMK were obtained after eliminating overlap.

### 3.2. AR-Related Target Genes

**Figure 3:**
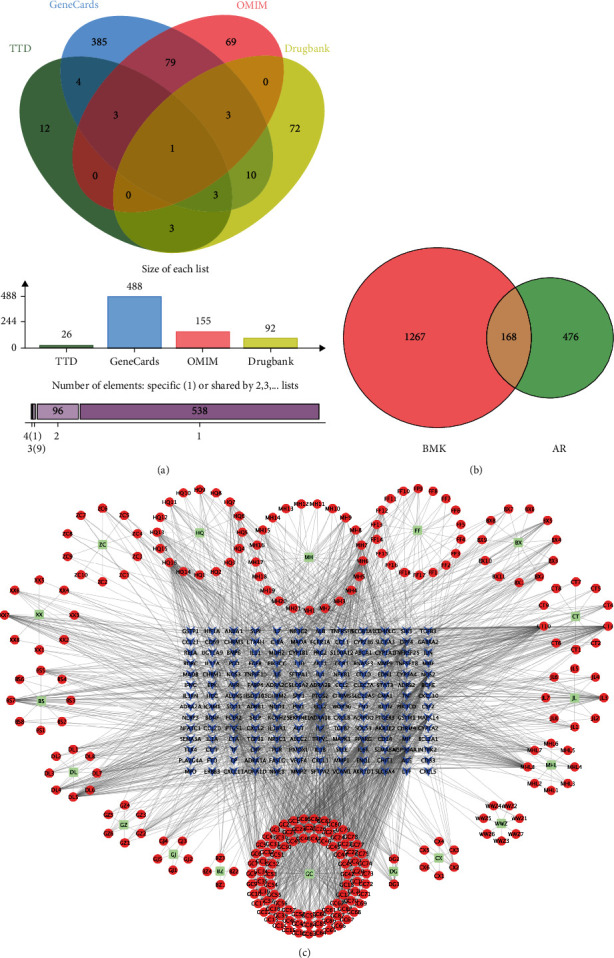
Network of components and targets. (a) VN diagram of AR disease-related target genes from 4 databases. (b) Statistical map of effective therapeutic target genes of BMK for AR. (c) Network diagram of the relationship between BMK active components and effective therapeutic target genes. The painting consists of two parts. The above table details the names of the ingredients corresponding to each node. In addition, the blue arrow in the middle shows the effective therapeutic target of BMK.

A total of 761 disease-related target genes were collected from four databases: TTD (26), DrugBank (82), GeneCards (488), and OMIM (155) (Figure 3(a)). After integration and removal of duplicates, 644 disease-related targets remained.

### 3.3. Construction of the Target Gene Network

A total of 168 overlapping target genes were obtained by integrating the data for the active components of BMK at AR targets (Figure 3(b)). Cytoscape software (version 3.7.2) was used to construct a component-target network from 168 effective therapeutic target genes corresponding to 244 active ingredients (Figure 3(c)).

### 3.4. Enrichment and Pathway Analysis for Effective Targets

**Figure 4:**
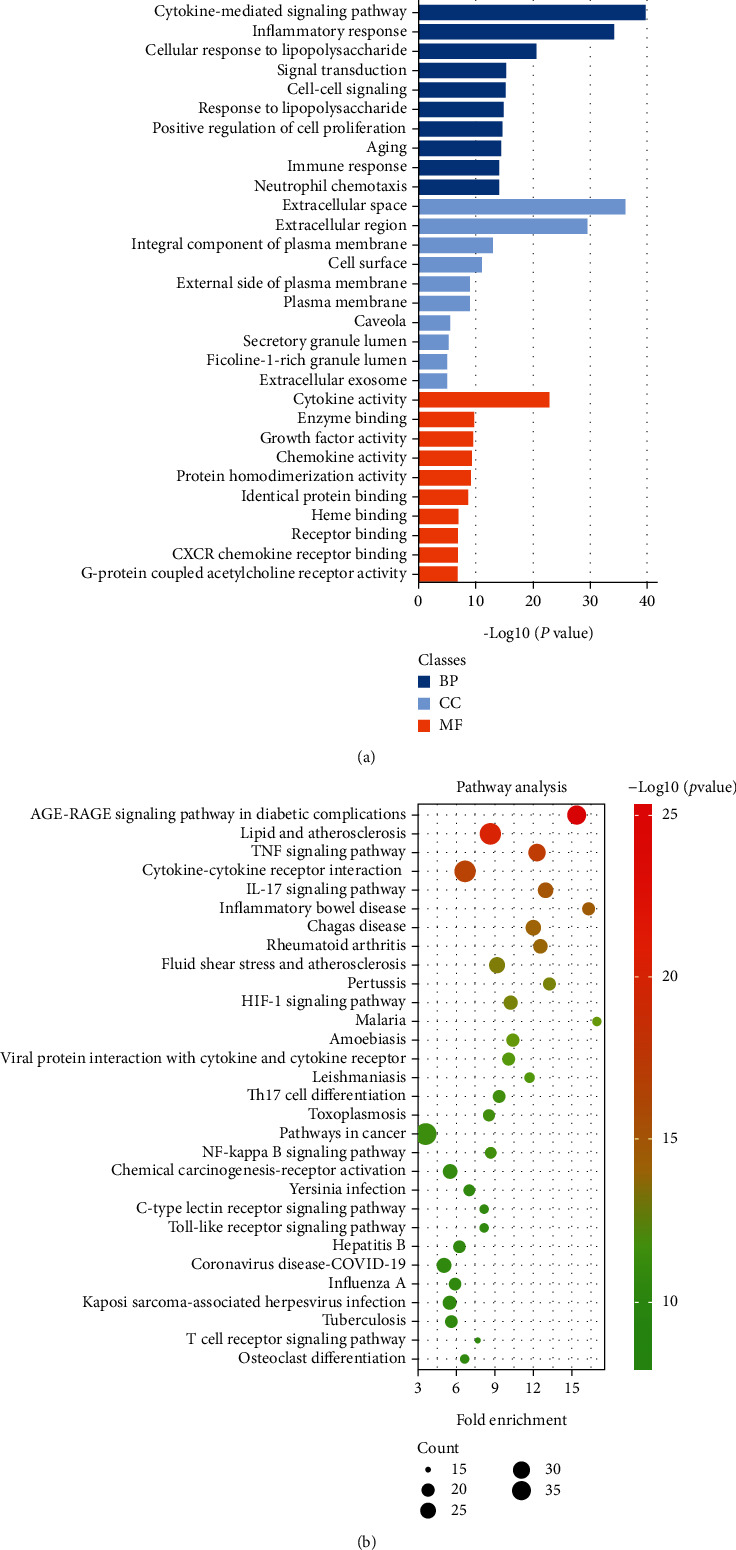
Enrichment analysis of effective target genes for BMK treatment of AR. (a) GO functional enrichment is derived from BP, CC, and MF. (b) In KEGG pathway enrichment, the top 30 pathways were determined according to *P* value <0.05.

Functional enrichment of 168 effective therapeutic target genes was performed using the DAVID database. In summary, GO pathway analysis indicated that the effective therapeutic targets exhibited cytokine activity, chemokine activity, and molecular functions such as growth factor activity. The analysis further revealed that these pathways included receptors involved in signal transduction, inflammation, immune responses, cell proliferation and aging, neutrophil chemotaxis, and other biological processes (Figure 4(a)). KEGG analysis revealed that the effective target genes were mainly related to inflammatory and immune diseases, cancer, cardiovascular diseases, and a variety of parasitic diseases. Th17 cell differentiation, hypoxia-inducible factor 1 (HIF-1), TNF, IL-17, NF-*κ*B, and Toll-like receptor signaling pathways were also involved (Figure 4(b)).

### 3.5. DEGs Obtained via RNA Sequencing

**Figure 5:**
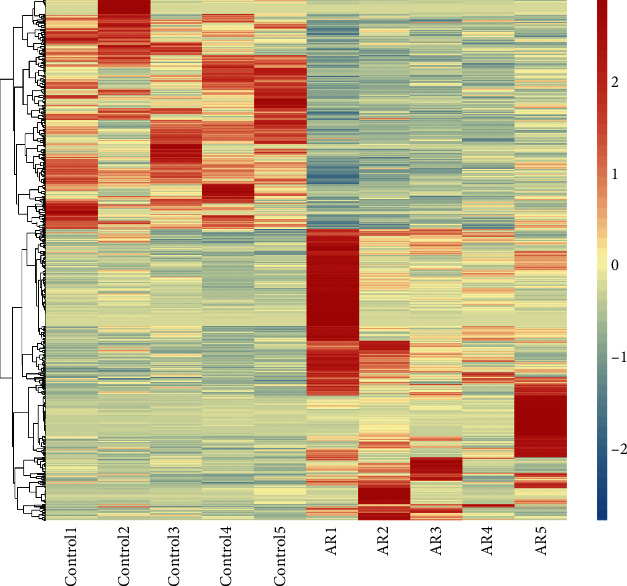
Heat map of DEGs obtained via RNA sequencing.

Pairwise comparisons of DEGs were performed between the control and AR groups. The results indicated that 34,787 genes were expressed in the control and AR groups. According to the criteria of FC up 1.5 times or down 1.5 times, *P* < 0.05 (both groups of FPKM values cannot be <1 at the same time), we identified 745, 329, and 416 DEGs of which were upregulated and downregulated, respectively. Edge*R* software was used to analyze DEGs between the control and AR groups, which are displayed as a heat map (Figure 5).

### 3.6. Enrichment Analysis of Key Target Genes

**Figure 6:**
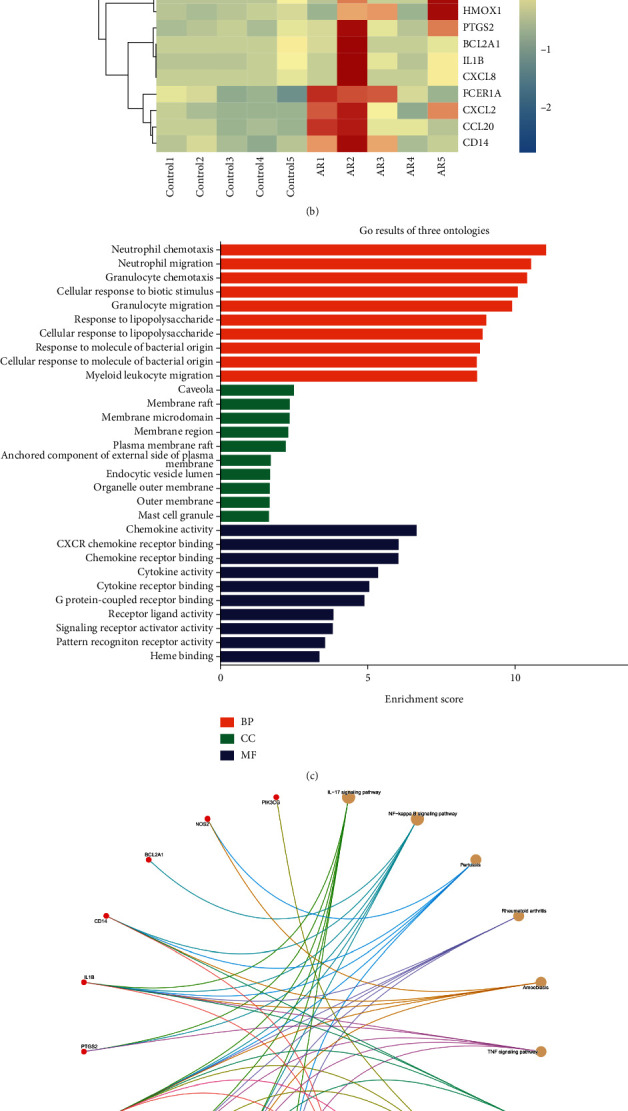
Key target genes. (a) VN diagram of network pharmacology and RNA-seq intersection. (b) Heat map of 20 key target genes. (c) GO functional enrichment is derived from BP, CC, and MF. (d) In KEGG pathway enrichment, the top 10 pathways were determined according to *P* value <0.05.

Effective targets from network pharmacology were combined with DEGs from RNA-seq, and overlapping target genes were analyzed using Edge*R* software to obtain 20 intersections as key target genes (Figure 6(a)). These target genes were further analyzed by Edge*R* software and are displayed as a heat map (Figure 6(b)). DAVID software was then used to analyze the 20 key target genes. Comprehensive analysis of the GO and KEGG enrichment results (Figures 6(c) and 6(d)) revealed that key target genes have cytokine, chemokine, and signal receptor activator activities, activate cytokines and chemokines through NF-*κ*B, TNF, and IL-17 signaling pathways, promote CXCR chemokine or cytokine binding to receptors, and participate in a variety of inflammatory or immune responses, including neutrophil chemotaxis and migration, rheumatoid arthritis or parasitic infection, and alcoholic liver disease.

### 3.7. Construction of the PPI Network and Identification of Hub Target Genes

**Figure 7:**
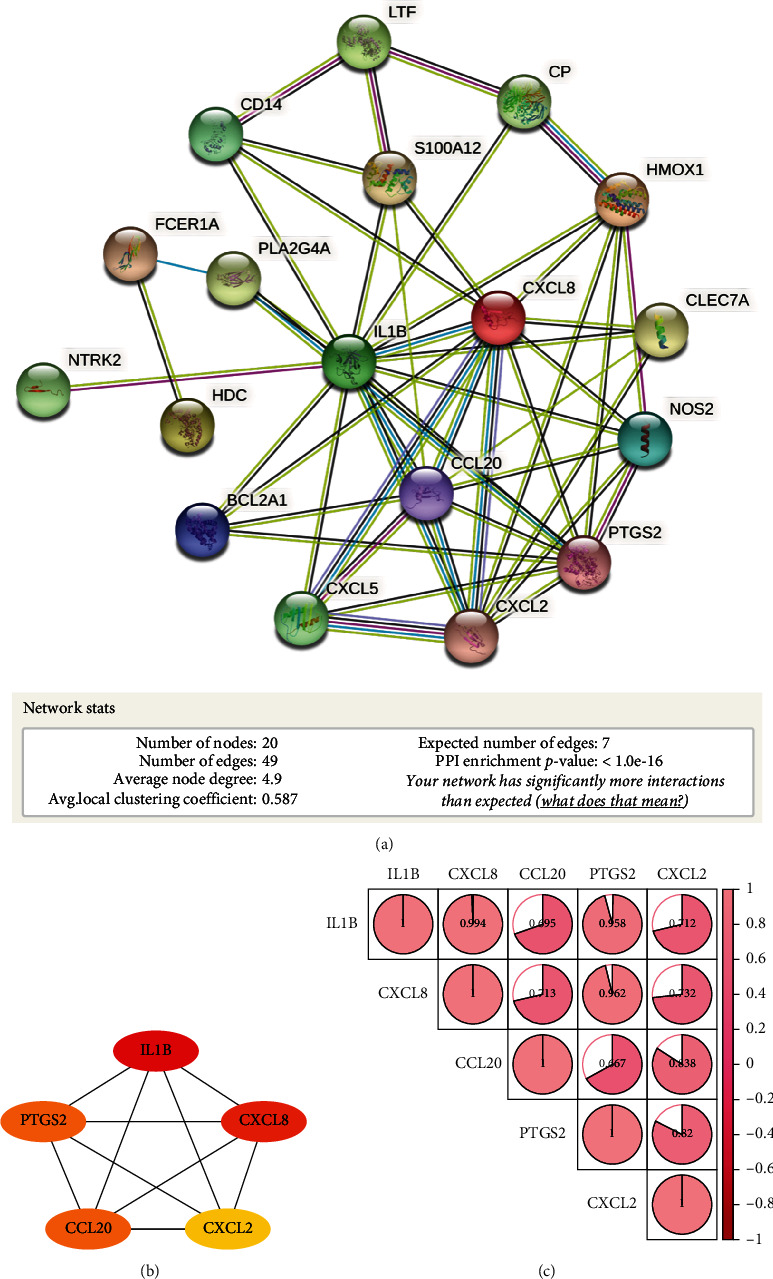
PPI network construction and identification of hub target genes. (a) PPI network construction of 20 key target genes. (b) 5 hub target genes. The degree value of each gene from yellow to red, with darker colors representing higher degree values. (c) Heat map of hub target gene correlation analysis.

The STRING database was used to create the PPI network diagram, which included 20 nodes and 49 edges (Figure 7(a)). Cytoscape (version 3.7.2) was then utilized for visual analysis. For topology analysis, Cytoscape software was utilized, and the findings of network parameters revealed that the median values of DC, CC, BC, and TC were 4.5, 0.523, 0.024, and 0.54, respectively. According to the greatest degree value, IL-1*β*, CXCL2, CCL20, CXCL8, and PTGS2 were chosen as hub target genes (Figure 7(b)). Then, we performed a correlation heat map analysis on 5 hub target genes the findings revealed that all target genes are positively associated (Figure 7(c)), and all genes are enriched in the NF-*κ*B signaling pathway (Supplementary. 1).

### 3.8. Molecular Docking

**Figure 8:**
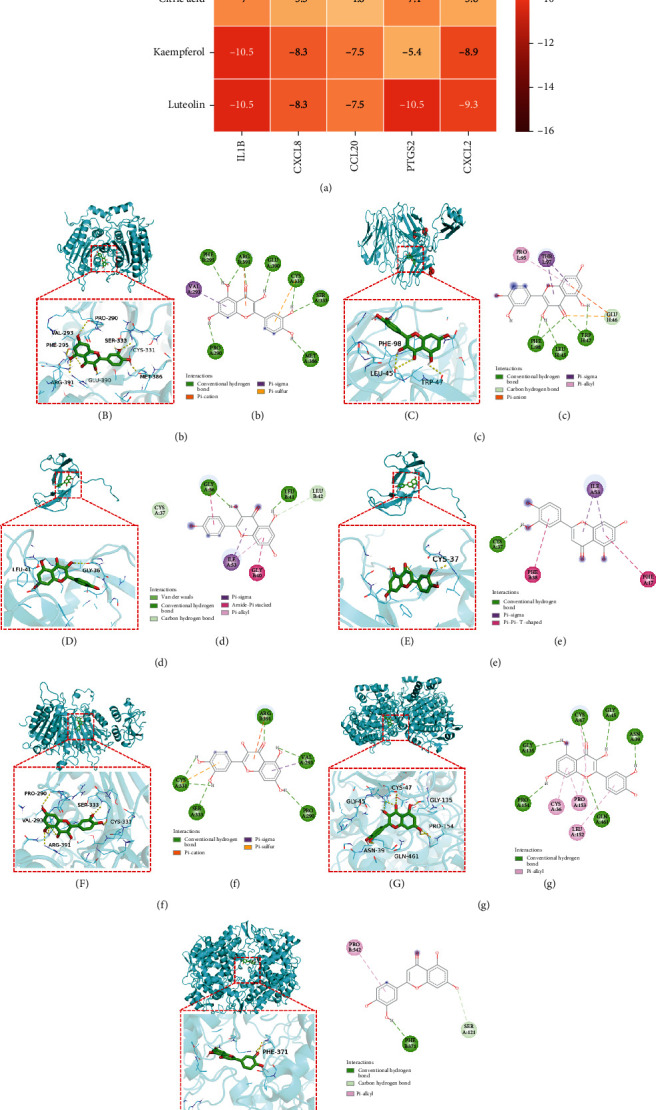
Molecular docking of effective component-hub target protein. (a) A heat map of affinity energy (kcal/mol). (b–h) shows the molecular docking 3D diagram. (B–H) shows the 2D diagram. Among them, (b-B) IL-1*β*-quercetin, (c-C) CXCL2-quercetin, (d-D) CXCL8-kaempferol, (e-E) CXCL8-luteolin, (f-F) CCL20-quercetin, (g-G) PTGS2-quercetin, and (h-H) PTGS2-luteolin.

To investigate the regulatory effect of BMK on hub target genes, we selected the five active ingredients with the highest degree values from the component-target network diagram for molecular docking (Table 3). AutoDock Vina program calculated the free binding energies between the active component and the hub target gene, and the results showed that the affinity energy ranged from -4.8 kcal/mol to -10.8 kcal/mol (Figure 8(a)). We generally consider that when the affinity energy of the receptor and the ligand is negative, this indicates that the two have binding activity. Strong binding activity is indicated when the affinity energy is less than -5 kcal/mol, and strong affinity energy is indicated when it is less than -7 kcal/mol [44]. This suggests that the five ligands are structurally stable and can spontaneously attach to IL-1*β*, CXCL2, CXCL8, CCL20, and PTGS2 receptors. Finally, we selected the effective ingredient with the best affinity activity to each hub target gene for molecular docking analysis (Figures 8(b)–8(h)).

Visual analysis was performed using PyMol and Discovery Studio software, and molecular docking results revealed that the interaction between small molecules of effective components and protein receptors was mostly dependent on conventional hydrogen, C-H, and Pi-bonds. Quercetin, for example, relies on seven hydrogen bonds to bind to PHE295, ARG391, GLU390, CYS331, SER333, MET386, and PRO290 at the IL-1*β* protein receptor junction. It forms three hydrogen bonds with the CXCL2 binding sites PHE98, LEU45, and TRP47. It forms five hydrogen bonds with the CCL20 binding sites CYS331,SER333, ARG391, VAL293, and PRO290. It forms six hydrogen bonds with the PTGS2 binding sites GLY135,CYS47, GLY45, ASN39, GLN461, and PRO154. Through two hydrogen bonds, kaempferol binds to GLY36 and LEU41 in CXCL8. Luteolin forms a single hydrogen bond with the CCXCL8 or PTGS2 binding sites CYS37 or PHE371, respectively.

### 3.9. Effect of BMK on Nasal Symptoms and Serum Total IgE Levels in AR Mice

**Figure 9:**
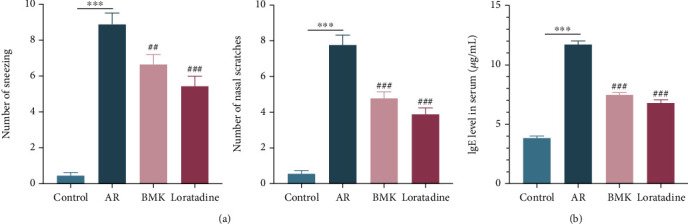
Effects of BMK on AR model mice. (a) Effects of BMK on sneezing and nasal scratches. (b) The expression level of IgE in serum was determined by ELISA. Compared with the control group, ^∗∗∗^*P* <0.001. Compared with the AR group, ^###^*P* < 0.001, ^##^*P* < 0.01.

To determine the degree to which BMK improves AR symptoms, AR model mice were treated with BMK or loratadine. In this study, the frequency of sneezing and nasal scratches was significantly higher in the AR group than in the control group (*P* < 0.001). After treatment, BMK effectively reduced the number of sneezing and nasal scratches and effectively relieved the severity of AR symptoms. There was no significant difference in the treatment effect between the loratadine and BMK groups (*P* > 0.05) (Figure 9(a)).

AR is caused by IgE-mediated inflammatory factor production and inflammatory cell infiltration. We evaluated serum total IgE levels to investigate BMK's anti-inflammatory effect on AR. Total IgE levels were considerably greater in the AR model group than in the control group, according to ELISA (*P* < 0.001). Levels of the IgE expression were significantly lower in the BMK and loratadine groups compared to the AR group (*P* < 0.001) (Figure 9(b)). Ultimately, BMK lowers blood total IgE levels during airway inflammation and so performs an immunomodulatory effect, which may explain its efficacy in reducing AR symptoms.

### 3.10. BMK Attenuates Pathological Changes Associated with AR

**Figure 10:**
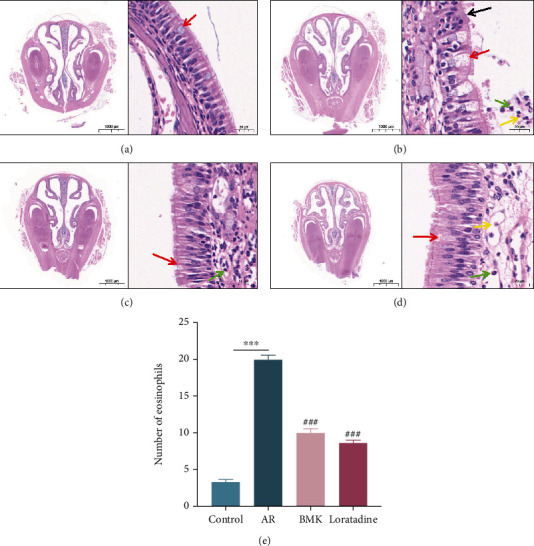
HE staining of nasal mucosa of mice. (a) represents the control group, (b) represents the AR model group, (c) represents the BMK group, and (d) represents the loratadine group. In each group, the intact nasal cavity structure was shown on the left (×10), and local staining was shown on the right (×400). Among them, red arrows mark goblet cells, black arrows mark cilia arrangement, yellow arrows mark neutrophils, and green arrows mark eosinophils. (e) The number of eosinophils in mice nasal mucosa tissue in different groups. Compared with the control group, ^∗∗∗^*P* < 0.001. Compared with the AR group, ^###^*P* < 0.001.

To further confirm the anti-inflammatory effect of BMK on AR, overall and local pathological changes were directly observed by staining nasal mucosa. HE staining revealed that the nasal cavity structure remained intact in each group. In the blank group, the epithelium of the nasal mucosa was pseudostratified in the ciliated columnar epithelium, with continuous and orderly cilia. The size of the mucosal lamina propria gland was normal, with no obvious vascular congestion or inflammatory cell infiltration (Figure 10(a)). In the AR group, the nasal mucosa exhibited a complex structure, disordered cellular arrangement, epithelial cilia damage, visible focal cilia detachment, and large numbers of inflammatory cell in the nasal cavity (Figure 10(b)). Mice in the BMK and loratadine groups exhibited an intact nasal mucosa, normal goblet cell volume, and reduced numbers of inflammatory cells (Figures 10(c) and 10(d)). The semiquantitative assessment of positive cells revealed that the number of eosinophils in the model group was substantially higher than in the control group (*P* < 0.001). When compared to the AR group, the number of eosinophils was considerably reduced in the BMK and loratadine groups (*P* < 0.001) (Figure 10(e)). These findings indicate that BMK significantly reduced inflammatory cell infiltration and effectively ameliorated pathological changes associated with AR in the nasal mucosa. These pathological findings were consistent with changes in nasal symptoms.

### 3.11. BMK Treats AR by Inhibiting the NF-*κ*B Signaling Pathway

**Figure 11:**
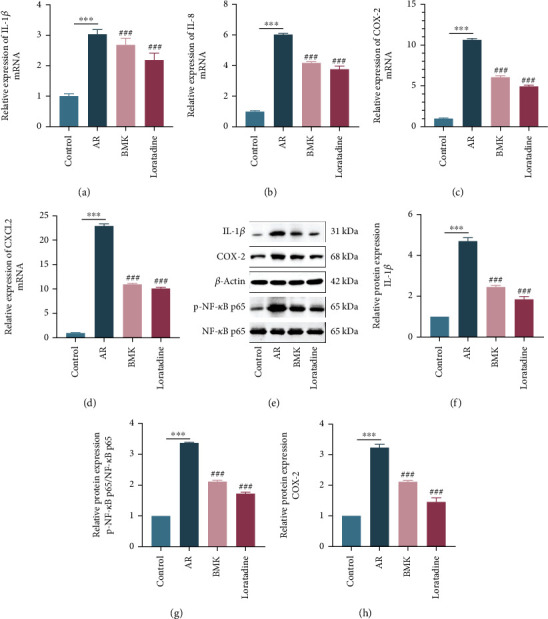
BMK treats AR by inhibiting the NF-*κ*B signaling pathway. (a–d) Effect of BMK on IL-1*β*, CXCL2, IL-8, and COX-2 mRNA expression levels in nasal mucosa of mice. (e–h) Western blotting was used to detect the protein expression of IL-1*β*, COX-2, NF-*κ*B p65, and p-NF-*κ*B p65 in nasal mucosa of mice.

The mRNA expression levels of IL-1*β*, IL-8, CXCL2, and COX-2 in mice nasal mucosa were evaluated using RT-qPCR in each group to investigate the mechanism through which BMK relieves AR. The results demonstrate that the mRNA expression levels of these indicators were considerably greater in the AR group than in the control group (*P* < 0.001). The mRNA expression of the targets was considerably decreased in the BMK and loratadine groups when compared to the AR model group (*P* < 0.001) (Figures 11(a)–11(d)).

To further identify the importance of BMK in the therapy of AR via regulating the NF-B signaling pathway, we assessed the levels of NF-*κ*B p65, p-NF-*κ*B p65, IL-1*β*, and COX-2 protein expression. Western blotting examination revealed the same pattern as mRNA expression level fluctuations (Figures 11(e)–11(h)). p-NF-*κ*B p65/NF-*κ*B p65 protein expression levels were vastly larger in the AR group than in the control group (*P* < 0.001). BMK and loratadine, on the other hand, substantially reduced these effects. These findings show that BMK has anti-inflammatory effects via inhibiting the activation of the NF-*κ*B signaling pathway, resulting in therapeutic effects.

## 4. Discussion

Abnormal climate change and increased environmental pollution continue to increase the risk of AR. In addition to their short duration of efficacy and long treatment cycle, common treatments for AR are associated with high drug tolerance and several side effects. Identifying a reliable alternative therapy is key to overcoming these issues in AR treatment. YPFS and XQLD have been widely used in the treatment of the immune system and respiratory diseases for thousands of years, especially asthma and AR. These Chinese herbal formulations contain multiple components that act on various target proteins to affect multiple pathogenic pathways. BMK, which combines YPFS and XQLD, has been shown to inhibit the secretion of proinflammatory cytokines and chemokines, thereby exerting clinically significant anti-inflammatory effects. However, the specific mechanism by which these effects occur remains to be elucidated.

Network pharmacology constructs ingredient-target-disease interaction networks by identifying multiple chemical components, targets, and signaling pathways, which can help reveal the pharmacodynamic mechanisms of TCM for the treatment of different diseases. Therefore, in this study, we used network pharmacology and RNA-seq to predict the active ingredients of BMK and the targets and signaling pathways for the treatment of AR. The results showed that validated targets from network pharmacology were combined with DEGs from RNA-seq, with a total of 20 intersections as key target genes. Enrichment analysis results revealed that key target genes have cytokine, chemokine, and signal receptor activator activities, activate cytokines and chemokines mainly through NF-*κ*B, TNF, and IL-17 signaling pathways, promote CXCR chemokine or cytokine binding to receptors, and are involved in a variety of inflammatory or immune responses. Through PPI network construction, 5 hub target genes (IL-1*β*, CXCL2, CCL20, CXCL8, and PTGS2) were finally identified, which are closely related to BMK treatment of AR.

A variety of cytokines and chemokines mediate the autoimmune response. IL-1*β*, a key proinflammatory cytokine produced primarily by monocytes and macrophages, activates the upregulation of adhesion molecules and promotes lymphocyte secretion and thus gets involved in the immune response [45]. Bachert et al. demonstrated that during the onset of AR, the level of the IL-1*β* expression in nasal secretions continues to rise, implying that IL-1*β* is involved in the entire inflammatory process [46]. Inflammatory chemokines include CXCL2 and IL-8. CXC chemotaxis is vital for neutrophil migration. CXCR2 is expressed by neutrophils and capabilities as a CXC chemokine receptor [47]. CXCL2 and IL-8 can indeed bind to CXCR2 receptors, recruit neutrophils to aggregation, and increase mucus secretion as well as the airway response, contributing to airway remodeling. Mast cells release inflammatory mediators that act on the nasal mucosa and cause nasal symptoms during the pathogenesis of AR. Prostaglandin synthesis, for instance, requires the catalytic action of COX-2 [48].The results indicate that restricting the COX-2 expression is especially critical for minimizing AR airway inflammation and improving nasal function.

In order to preliminarily verify the effectiveness of BMK, 5 active ingredients were selected for molecular docking with 5 hub target genes. The results showed that these small molecules could freely bind to the target protein, indicating that BMK could play a certain therapeutic role. Jafarinia et al. and Mlcek et al. showed that quercetin exerts anti-inflammatory and immunomodulatory effects in allergic diseases by reducing IgE expression levels, regulating Th1/Th2 stability and inhibiting histamine production [49, 50]. Jang et al. demonstrated that luteolin could alleviate inflammatory cell infiltration and exert antiallergic effects in mouse models of allergic asthma and allergic rhinitis [51]. In both clinical and animal studies, Liang et al. revealed that luteolin relieved allergic rhinitis by decreasing IL-4 production [52]. Oh et al. developed a mouse model of allergic rhinitis and showed that kaempferol had antiallergic effects through modulating IL-32 and TSLP production as well as caspase-1 activity [53]. Deoxyandrographolide and citric acid, on the other hand, exhibit anti-inflammatory and immunomodulatory properties [54, 55]. The findings presented above provide a theoretical foundation for further demonstrating BMK's potential therapeutic role and significance in AR.

In the pathogenesis of AR, IgE binds to the Fc*ε*RI on mast cells and other surfaces. Mast cells can release a variety of mediators when reexposed to allergens and simultaneously recruit and activate effector cells, thereby mediating inflammatory responses Continuous secretion of inflammatory factors leads to increased vascular permeability and exacerbates the inflammatory response of the upper and lower airways, further leading to nasal and bronchial mucosal edema, aggravating inflammatory cell infiltration, and ultimately leading to immune dysfunction and induced acute airway hyperreaction. Our experimental study found that the secretion levels of IgE in the serum were significantly higher in AR model mice than in control mice. Moreover, treatment with BMK significantly attenuated this increase in the IgE expression. The severity of allergic behaviors in AR mice, including sneezing and nasal scratching, was directly influenced by pathological changes. According to our analysis, BMK treatment significantly improved AR symptoms. In addition, HE staining verified that BMK reduced the release of inflammatory mediators, improved ciliated epithelium activity and integrity, reduced airway edema and mucus secretion, and relieved inflammatory infiltration. These findings indicate that BMK exerts antiallergic effects by inhibiting the secretion of inflammatory cells and reducing airway inflammation.

Network pharmacology analysis revealed that all five central target genes obtained from PPI network construction were enriched in the NF-*κ*B pathway. Therefore, we suggest that modulation of the NF-*κ*B signaling pathway is the main mechanism of action of BMK in the treatment of AR. To better study the mechanism of BMK, we first looked at the mRNA expression levels of cytokines and chemokines. The results of the RT-qPCR detection divulged that BMK effectively reduced the mRNA expression levels of IL-1*β*, CXCL2, IL-8, and COX-2. What is more, the expression of various proinflammatory cytokines and chemokines is completely reliant on transcription factor activation [56]. NF-*κ*B can be found in nearly all animal cell types. NF-*κ*B p65 regulates DNA transcription and cytokine synthesis and is involved in inflammatory and immunological responses. Of course, NF-*κ*B p65 phosphorylation regulates the transcriptional role of downstream components. Protein expression levels of NF-*κ*B p65, p-NF-*κ*B p65, IL-1*β*, and COX-2 were measured to better understand the mechanism of action. BMK suppressed the phosphorylation of NF-*κ*B p65, lowered its transcriptional activity, and decreased the expression of inflammatory cytokines and chemokines.

## 5. Conclusion

In this study, we explored the potential targets of BMK in the treatment of AR using network pharmacology, transcriptomics, and bioinformatics analyses. Our experiments verified that BMK reduces the expression of inflammatory cytokines and chemokines by inhibiting the NF-*κ*B signaling pathway and transcription factor activation, thereby reducing airway inflammation in AR mice. Results from animal experiments are consistent with our previous clinical studies. However, this study was only conducted *in vivo*, and further verification of the predicted results *in vitro* is required. Despite these limitations, our study provides a theoretical basis for the clinical efficacy of BMK in the context of AR, which may aid in the development of simpler, more effective treatment strategies.

## Figures and Tables

**Table 1:** BMK consists of 18 herbal medicines.

Chinese name	Latin name	Abbreviation	Function
Huang Qi	Astragalus aboriginum Spreng	HQ	Improve immunity, antiviral
Bai Zhu	Atractylodes macrocephala Koidz	BZ	Enhance immunity and adrenal cortex function
Fang Feng	Saposhnikovia divaricata (Trucz.) Schischk	FF	Anti-inflammatory, antibacterial, enhance immunity
Xi Xin	Asarum sieboldii Miq.	XX	Anti-inflammatory, antiallergic, immune regulation
Ban Xia	Pinellia ternata (Thunb.) Makino	BX	Relieve cough and expectorant
Wu Wei Zi	Schisandra chinensis (Turcz.) Baill	WWZ	Antiallergy, antibacterial, enhance immunity
Gan Jiang	Zingiber officinale Rosc	GJ	Anti-inflammatory, disease-resistant microbial, promote immunity
Gui Zhi	Cinnamomum cassia (L.) J.Presl	GZ	Antiallergic, anti-inflammatory
Ma Huang	Ephedra sinica Stapf	MH	Reduce mucous edema, inhibit the release of allergenic media
Bai Shao	Cynanchum otophyllum C.K.Schneid	BS	Antibacterial and anti-inflammatory
Di Long	Pheretima	DL	Antihistamines
Chuan Xiong	Ligusticum wallichii Franch	CX	Enhances immunity and relieves airway spasm
Chan Tui	Cicadae Periostracum	CT	Antiallergic, anti-inflammatory
Mo Han Lian	Eclipta alatocarpa Melville	MHL	Modulates immune
Ji Li	Tribulus terrestris L.	JL	Antiallergic
Zi Cao	Lithospermum erythrorhizon Sieb. et Zucc	ZC	Anti-inflammatory, antibacterial
Dang Gui	Angelica sinensis (Oliv.) Diels	DG	Enhance immunity
Gan Cao	Glycyrrhiza uralensis Fisch	GC	Antiallergy, increase corticosteroids

**Table 2:** RT-qPCR primer sequences.

Target gene	Primer name	Sequence 5′⟶3′
*β*-Actin	Mouse-*β*-actin-F	CACGATGGAGGGGCCGGACTCATC
	Mouse-*β*-actin-R	TAAAGACCTCTATGCCAACACAGT
IL-1*β*	Mouse-IL-1*β*-F	TCAGGCAGGCAGTATCACTC
	Mouse-IL-1*β*-R	AGCTCATATGGGTCCGACAG
IL-8	Mouse-IL-8-F	TTCATTCCTCTCAAACTCA
	Mouse-IL-8-R	AAACAAATCATACTCCCAT
COX-2	Mouse-COX-2-F	ATCATAAGCGAGGACCTGGG
	Mouse-COX-2-R	TCAGGGATGTGAGGAGGGTA
CXCL2	Mouse-CXCL2-F	AAGAACATCCAGAGCTTGAGTGT
	Mouse-CXCL2-R	GCCTTGCCTTTGTTCAGTATCTT

**Table 3:** Key protein receptor and small molecule ligand information in molecular docking.

Target	PBD ID	Center coordinates	Compound Mol ID	PubChem ID	Compound	Molecular formula
IL-1*β*	3E4C	-1.891, -8.649, -3.74	SMIT00105	311	Citric acid	C_6_H_8_O_7_
CXCL8	6I31	10.542, 26.563, 1.843	SMTT11560	21679042	Deoxyandrographolide	C_20_H_30_O_4_
CCL20	1M8A	2.981, -2.117, -3.049	MOL000098	5280343	Quercetin	C_15_H_10_O_7_
PTGS2	3NT1	-33.195, -39.47, -43.669	MOL000006	5280445	Luteolin	C_15_H_10_O_6_
CXCL2	5OB5	-24.872, 26.569, -5.476	MOL000422	5280863	Kaempferol	C_15_H_10_O_6_

## Data Availability

The datasets generated for this study can be obtained from the corresponding authors upon reasonable request.

## Abbreviations

AR: Allergic rhinitis
IgE: Immunoglobulin E
AIT: Allergen-specific immunotherapy
SCIT: Subcutaneous immunotherapy
SLIT: Sublingual immunotherapy
BMK: Bimin Kang Mixture
YPFS: Yupingfeng San
XQLD: Xiaoqinglong Decoction
Fc*ε*RI: High-affinity immunoglobulin E receptor
AHR: Aryl hydrocarbon receptor
MUC5AC: Mucin 5 subtype AC
MUC5B: Mucin 5 subtype B
TNF-*α*: Tumor necrosis factor alpha
DEGs: Differentially expressed genes
TCMSP: Traditional Chinese Medicine Systems Pharmacology Database and Analysis Platform
OB: Oral bioavailability
DL: Drug-likeness
TTD: Therapeutic Target Database
OMIM: Online Mendelian Inheritance in Man
PPI: Protein–protein interactions
CC: Closeness centrality
BC: Betweenness centrality
DC: Topological coefficient
TC: Degree centrality
GO: Gene Ontology
KEGG: Kyoto Encyclopedia of Genes and Genomes
FC: Fold change
CC: Cellular component
BP: Biological process
MF: Molecular function
DAVID: Database for Annotation, Visualization, and Integrated Discovery
PBS: Phosphate-buffered saline
HE: Hematoxylin and eosin
ELISA: Enzyme-linked immunosorbent assays
RT-qPCR: Real-time quantitative polymerase chain reaction
IL-1*β*: Interleukin-1B
CXCL8: C-X-C motif chemokine ligand 8
CXCL2: C-X-C motif chemokine ligand 2
PTGS2: Prostaglandin-endoperoxide synthase 2.

## Supplementary Materials

Supplementary Materials Supplementary File 1: the NF-*κ*B signaling pathway.
