# Supplementary figures and images for: Molecular Mechanisms Underlying the Effects of Bimin Kang Mixture on Allergic Rhinitis: Network Pharmacology and RNA Sequencing Analysis
Source: Biomed Res Int. 2022 Oct 28;2022:7034078. doi: 10.1155/2022/7034078 (PMC9635970; doi:10.1155/2022/7034078)

## NF-KAPPA B SIGNALING PATHWAY

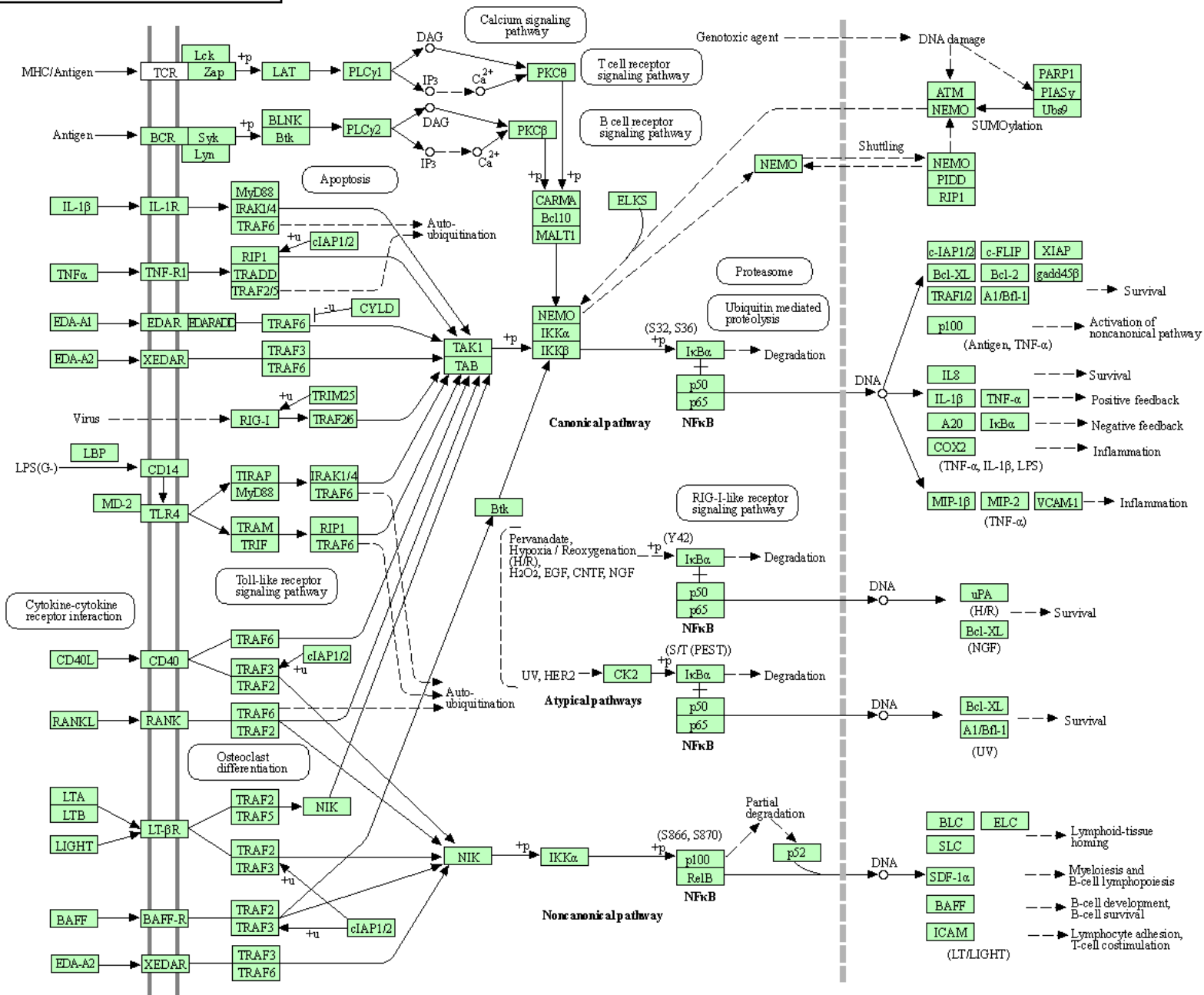

Supplement: Supplementary Materials — Supplementary File 1: the NF-κB signaling pathway. [file 7034078.f1.pdf]
